# Effect of High-Fructose Diet-Induced Metabolic Syndrome on the Pituitary-Gonadal Axis in Male Rats

**DOI:** 10.3390/biomedicines10123009

**Published:** 2022-11-23

**Authors:** Shih-Min Hsia, Yi-Fen Chiang, Hsin-Yuan Chen, Mohamed Ali, Paulus S. Wang, Kai-Lee Wang

**Affiliations:** 1 School of Nutrition and Health Sciences, College of Nutrition, Taipei Medical University, Taipei 11031, Taiwan; 2 Graduate Institute of Metabolism and Obesity Sciences, College of Nutrition, Taipei Medical University, Taipei 11031, Taiwan; 3 School of Food and Safety, College of Nutrition, Taipei Medical University, Taipei 11031, Taiwan; 4 Nutrition Research Center, Taipei Medical University Hospital, Taipei 11031, Taiwan; 5 TMU Research Center for Digestive Medicine, Taipei Medical University, Taipei 11031, Taiwan; 6 Clinical Pharmacy Department, Faculty of Pharmacy, Ain Shams University, Cairo 11566, Egypt; 7 Department of Physiology, School of Medicine, National Yang Ming Chiao Tung University, Taipei 112, Taiwan; 8 Medical Center of Aging Research, China Medical University Hospital, Taichung 404328, Taiwan; 9 Department of Biotechnology, College of Health Science, Asia University, Taichung 413305, Taiwan; 10 Department of Nursing, Ching Kuo Institute of Management and Health, Keelung 20301, Taiwan

**Keywords:** diabetes, testosterone, Leydig cells, hypothalamus-pituitary-gonadal (HPG) axis

## Abstract

Plasma testosterone levels have been found to decrease in older insulin-resistant male patients. Both lower total testosterone levels and a higher incidence of metabolic syndrome have also been reported. The aim of this study was to investigate the effects of high-fructose diet-induced diabetes on both the testosterone release by Leydig cells and the activity of the hypothalamus–pituitary–gonadal (HPG) axis in male rats. Male rats were fed with either standard chow (control group) or a high-fructose diet (fructose-fed group) for 21 weeks. Hyperglycemia, hyperinsulinemia, and hypertension were observed in the fructose-fed group. Moreover, plasma testosterone and LH levels decreased in the fructose-fed group compared to the control group. Sperm motility was also reduced by 15% in the fructose-fed rats. In contrast, the basal release of testosterone from rat Leydig cells was not altered by fructose feeding. Moreover, in vitro studies showed that the testosterone release, in response to different stimulants, including forskolin (an adenylyl cyclase activator, 10^−5^ M), 8-Br-cAMP (a permeable analog of cAMP, 10^−5^ M), A23187 (a calcium ionophore, 10^−5^ M), or 25-hydroxy-cholesterol (water-soluble cholesterol, 10^−5^ M), did not significantly differ between the fructose-fed and control groups. Interestingly, the release of testosterone in response to human chorionic gonadotropin (hCG, 0.05 IU/mL) was enhanced by eightfold in the control group, but elevenfold in the fructose-fed group. LH receptor expression in rat Leydig cells was also increased. Moreover, LH secretion from the anterior pituitary was altered in the fructose diet-fed group. These results suggest that fructose diet-fed rats have lower plasma testosterone levels, which can lead to a higher sensitivity of hCG in Leydig cells.

## 1. Introduction

Diabetes mellitus (DM), a metabolic disorder characterized by chronic hyperglycemia, derives from either a lack of or resistance to insulin [1]. DM and obesity affect more than a third of the population worldwide. By 2021, the global prevalence of diabetes among people aged 20–79 was estimated at 536.6 million (10.5%), and the rates will increase persistently. DM is the seventh-leading cause of death globally as it causes damage to a range of organs/systems in the human body, such as the heart, blood vessels, and kidneys [2].

The hypothalamic–pituitary–gonadal (HPG) axis plays a critical role in the development and regulation of the reproductive system. The gonadotropin-releasing hormone (GnRH), a hormone secreted from the hypothalamus, stimulates luteinizing hormone (LH) production from the anterior pituitary, thereby stimulating androgen synthesis and secretion from Leydig cells. Androgen is required for the spermatogenesis and maturation of secondary sexual characteristics. Male infertility affects approximately 7% of all men, and is mainly characterized by azoospermia or HPG axis alterations [3].

Diabetes is linked to the impairment of male reproductive function. There is a bidirectional relationship between lower testosterone levels and insulin resistance status [4,5]. Moreover, subfertility is highly prevalent (51%) in diabetic patients [6]. A large proportion (30–40%) of patients with type 2 diabetes (T2DM) suffer from hypogonadism [7]. Higher erectile dysfunction was also found in men with T2DM [8]. In addition, reduced sperm quality and motility were found in diabetic men. Mechanistically, there are more sperm abnormalities/DNA damage in diabetics [9]. Furthermore, diabetes and obesity cause male infertility through reduced testosterone levels and sperm parameters, including sperm morphology, concentration, and motility [10,11,12].

Low androgen caused by diabetes may be related to the occurrence of other diseases. For example, hypogonadal males with diabetes have a higher plaque score, coronary artery disease, and cardiovascular mortality risk [13,14], which might be due to hyperlipidemia induced by lower androgen levels [15]. Additionally, hypogonadism is also associated with chronic kidney disease which mainly occurs as a consequence of diabetes [16,17]. Therefore, it is important to study reproductive disorders caused by diabetes because it might be associated with damage caused to other systems as well.

Previous studies have shown an inverse association between diabetes and male fertility. Moreover, this effect is related to the disturbance of the HPG axis. However, the mechanisms of the effects of diabetes on the HPG axis are still obscure. The purpose of the present study was to evaluate the effects of a high-fructose diet on the HPG axis in rats with metabolic syndrome.

## 2. Materials and Methods

### 2.1. Animals

Five-week-old male Sprague–Dawley rats were purchased from BioLasco Co., Ltd. and adapted for one week by housing in a temperature-controlled room (22 ± 1 °C) with 14 h of artificial illumination daily (600–2000 h) and ad libitum access to water and food. Thereafter, rats were randomly divided into two groups to be fed on either a 65% high-fructose diet or a standard chow diet for 21 weeks [18,19]. Hyperinsulinemia and hyperglycemia were found in the high-fructose diet group. Hyperinsulinemia was found in the high-fructose diet animals (plasma insulin levels were 70.63 ± 9.76 and 102.01 ± 23.15 μU/mL*h in the control and high-fructose diet groups, respectively). All animal procedures conducted in this study were based on a protocol approved by the Institutional Animal Care and Use Committee (IACUC) (LAC-2018-0034) of Taipei Medical University.

### 2.2. Materials

We obtained [^3^H]-testosterone from Amersham Pharmacia Biotech (Bucks, UK). All the other chemicals were purchased from Sigma–Aldrich Chemical Co. (St. Louis, MO, USA). The doses of drugs are being as the final molar concentrations used in the flask.

### 2.3. Preparation of Rat Leydig Cells

Animals were sacrificed, and the testes were decapsulated. Leydig cells were isolated and digested by collagenase and Percoll separation, as our previous study described [20,21]. In brief, testicular interstitial cells were isolated after collagenase digestion and centrifugation. Thereafter, cells were loaded to the upper layer of the continuous Percoll gradient and centrifuged. Leydig cells, located at a 3–7 mL layer from the bottom after centrifugation, were collected and washed twice to remove Percoll. The cell concentration (2 × 10^5^ cells/mL) and viability (over 95%) were determined by using a hemocytometer and the trypan blue method. Approximately 87% of Leydig cells were found after 3β-hydroxysteroid dehydrogenase (3β-HSD) staining.

### 2.4. Evaluation of Basal and Evoked Testosterone Release by Rat Leydig Cells

Rat Leydig cells (1 × 10^5^ cells/tube) were incubated in a 1% (*w*/*v*) BSA-M199 medium for 60 min at 34 °C under 95% O_2_–5% CO_2_. Different stimulators including human chorionic gonadotropin (hCG, 0.05 IU/mL), 8-Br-cAMP (a membrane-permeable analog of cyclic AMP, 10^−4^ M), and forskolin (an adenylyl cyclase activator, 10^−5^ M) were used to evaluate the alteration of LH and cAMP/PKA signaling pathway in rat Leydig cells after fructose feeding. Furthermore, different precursors, including 25-hydroxycholesterol (25-OH-C, (a membrane permeable cholesterol, 10^−5^ M), pregnenolone (P_5_, 10^−5^ M), progesterone (P_4_, 10^−5^ M), and androstenedione (AND, 10^−6^ M) were used to evaluate the activity of steroidogenesis enzymes.

### 2.5. Testosterone RIA

We used a radioimmunoassay (RIA) (sensitivity 10 pg/mL, intra-assay coefficients of variation (CV) 4.1%, inter-assay 4.7%) to determine plasma and media testosterone levels [20,21].

### 2.6. Sperm Count and Vitality Assay

The sperm vitality was evaluated according to our previous study [21]. The rat cauda epididymis was cut into two pieces and incubated with gentle shaking in 1% BSA-M199 medium for 10 min at 34 °C. After gently shaking for 10 min, the sperm was dissociated into the medium. The sperm suspension was mixed with 0.4% Trypan blue solution. The numbers of active and quiescent sperms were counted by using a hemocytometer under a microscope.

### 2.7. Western Blot Analysis

The protein expressions of LHR (Santa Cruz Biotechnology, 1:1000 dilution) and β-actin (Chemicon, Temecula, CA, USA, 1:8000 dilution) in rat Leydig cells (2 × 10^6^ cells/tube) were evaluated by Western blotting according to previous described methods [20,22]. Rat Leydig cells were homogenized with buffer (1.5% Na-lauroyl sarcosine, 2.5 mM Tris base, 1 mM EDTA, and 0.1% PMSF, pH 7.8). Then, 20 μg of protein was denatured by boiling, separated according to molecular weight by 10 or 12% electrophoresis, and then transferred onto polyvinyl difluoride membranes (NEN Life Science Products, Boston, MA, USA). The membranes were then blocked with 5% (*w*/*v*) nonfat dry milk in TBST buffer for 2 h in RT. Thereafter, membranes were incubated with specific primary antibodies overnight at 4 °C. After being washed three times, membranes were incubated with horseradish peroxidase-conjugated goat anti-rabbit antibodies for 2 h in RT. Protein levels were detected by chemiluminescence.

### 2.8. Statistical Analysis

Data were presented as the mean ± standard error of the mean (SEM) and used the Shapiro–Wilk test to test the normality. In the normal distribution condition, differences among all groups were assessed by one-way analysis of variance (ANOVA), followed by Duncan’s multiple-range test, which was used for comparison among different treatment groups. A Student’s unpaired *t*-test was used to compare the control and fructose-fed groups. The data did not pass the normality test, which used Mann–Whitney U to compare the control and fructose-fed groups. The significance was set at *p* < 0.05.

## 3. Results

### Effects of Fructose-Fed Diet on Plasma Testosterone Levels

**Figure 1:**
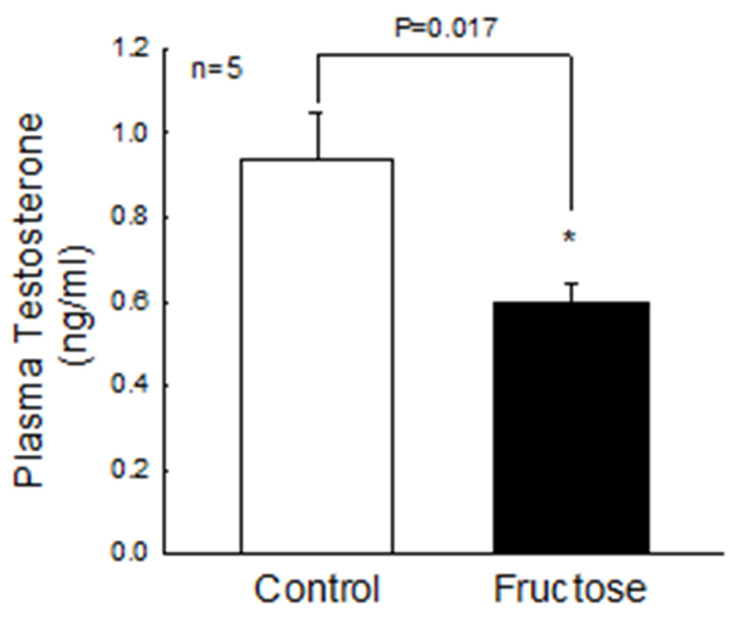
Effects of the fructose-fed diet on plasma testosterone levels. Plasma testosterone levels in the control and fructose-fed group. * *p* < 0.05 as compared to the control group. Each value presents mean ± SEM. Similar results were repeated twice.

**Figure 2:**
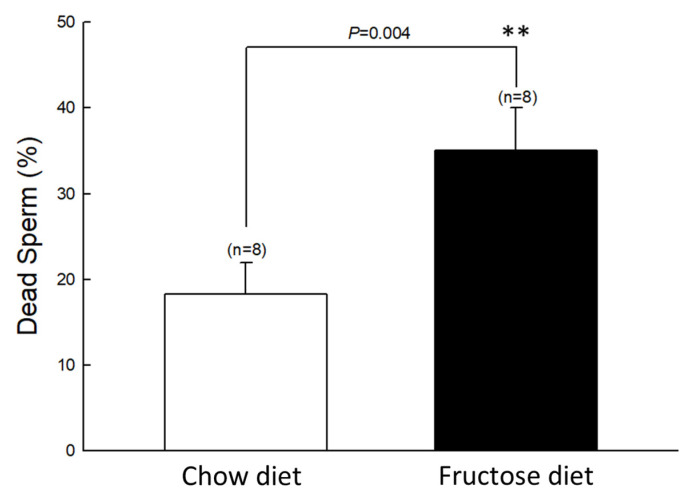
Effects of the fructose diet-fed on sperm motility. Semen was collected from the epididymis and put on the warmed slides; thereafter, the percentage of sperm motility was examined. ** *p* < 0.01 as compared to the control group (white column). Each value presents mean ± SEM. Similar results were repeated twice.

We first assessed whether a high-fructose diet could affect plasma androgen concentrations. The experimental results are shown in Figure 1. After 21 weeks of a high-fructose diet, the mean plasma testosterone level was 0.6 ng/mL, which was significantly lower than that of the control group (0.93 ng/mL, *p* = 0.017). Moreover, the percentage of dead sperm was significantly increased in the fructose-fed group as compared to the control group (Figure 2). These results imply that the high-fructose diet caused damage to the male reproductive system.

**Figure 3:**
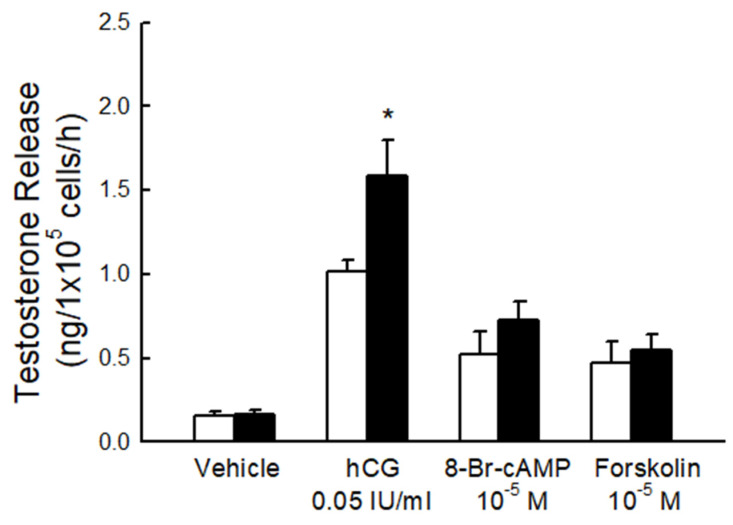
Testosterone release in response to different stimulators. Basal and evoked testosterone release in response to hCG, 8-Br-cAMP, or forskolin in rat Leydig cells purified from the fructose-diet fed group (black column) and control group (white column). * *p* < 0.05 as compared to the control group. Each value presents mean ± SEM. Similar results were repeated twice.

Furthermore, the testosterone secretion capacity of primary rat Leydig cells was evaluated (Figure 3). Interestingly, testosterone release, in response to hCG (0.05 IU/mL), was increased significantly in rat Leydig cells and purified from the fructose-fed group compared to the normal group (Figure 3). The hCG dosage is enough to induce the maximum steroidogenic response, as shown in our previous experimental studies [20,21]. To determine whether the fructose-rich diet also can affect the cAMP/PKA pathway, the main downstream mechanism of the LH receptor, the cAMP analog (8-Br-cAMP), and the PKA activator (forskolin) were utilized at 10^−5^ M. A nonstatistically significant elevation of testosterone release was found between the two groups in response to 8-Br-cAMP and forskolin. These results suggest that elevation mainly occurs due to the change in LH sensitivity (hypersensitivity).

**Figure 4:**
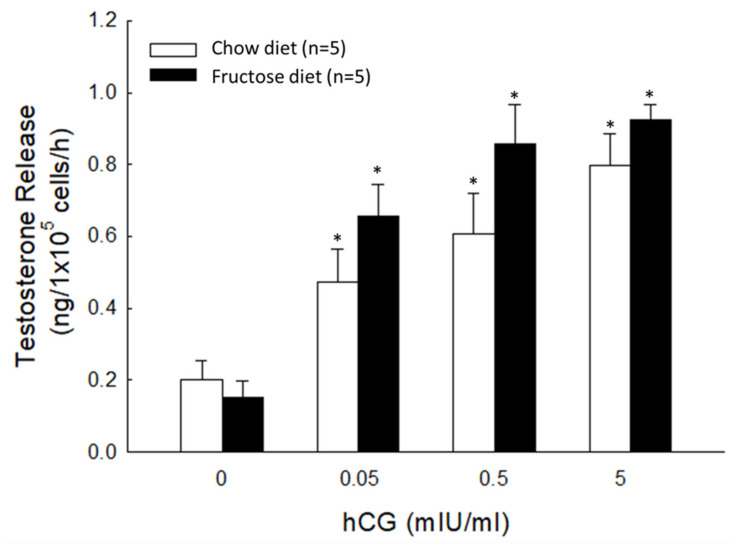
Testosterone release in response to different concentrations of hCG. Basal and evoked testosterone release in response to different concentrations of hCG in rat Leydig cells purified from the fructose-diet fed group (black column) and the control group (white column). * *p* < 0.05 as compared to the vehicle group (dosage:0). Each value presents mean ± SEM. Similar results were repeated twice.

To further evaluate whether the fructose-rich diet can affect LH sensitivity in response to hCG in vitro, rat Leydig cells were purified and then treated with different concentrations of hCG (including 0.05, 0.5, and 5 mIU/mL) (Figure 4). As our data show, there was a no significant stimulation of diabetes on Leydig cell responsiveness to hCG. This result is consistent with the results presented in Figure 3. 

**Figure 5:**
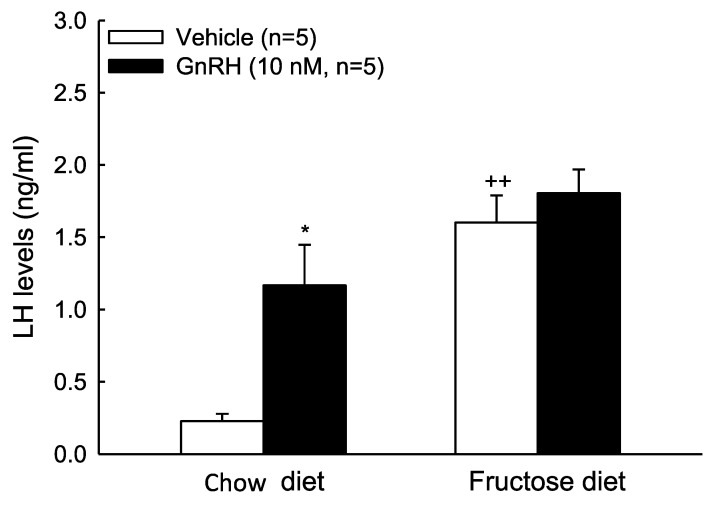
Effects of fructose-fed diet on LH release. Basal and GnRH-evoked LH release in rat AP tissues isolated from fructose-diet fed group (black column) and control group (white column). * *p* < 0.05 as compared to the control group. ^++^ *p* < 0.01 as compared to the chow diet group. Each value presents mean ± SEM. Similar results were repeated twice.

Furthermore, we further evaluated the LH secretion ability of the anterior pituitary isolated from the two groups. Higher basal LH secretion was found in the fructose diet group. In contrast, there was a lower LH secretion ability in response to GnRH (10 nM) administration compared to the control group (Figure 5). These results suggest that the disorder of the HPG axis and the impairment of the male reproductive system caused by the high-fructose diet can mainly be attributed to the effect on the secretion of LH from the anterior pituitary (secondary or/and tertiary defect).

**Figure 6:**
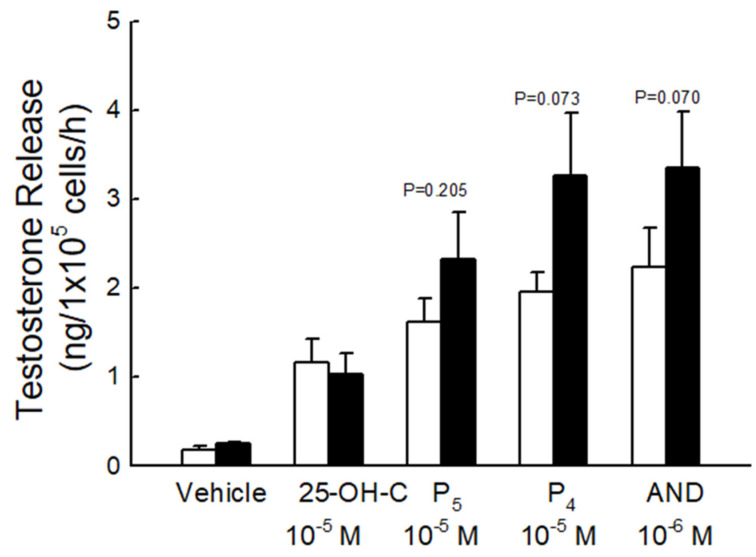
Testosterone release in response to different precursors. Basal and evoked testosterone release in response to different precursors in rat Leydig cells purified from the fructose-diet fed group (black column) and the control group (white column). Testosterone was determined after treating Leydig cells with different precursors including 25-hydroxycholesterol (25-OH-C), pregnenolone (P_5_), progesterone (P_4_), and androstenedione (AND) for 1 h. Each value presents mean ± SEM. Similar results were repeated twice.

In order to study the activity of key steroidogenic enzymes in the two groups, testosterone levels were evaluated after incubating rat Leydig cells with different precursors (Figure 6). The testosterone secretions were increased after incubation with different precursors. Although the activity of these enzymes increased in the fructose-fed group as compared to the control group, this result was not significant.

**Figure 7:**
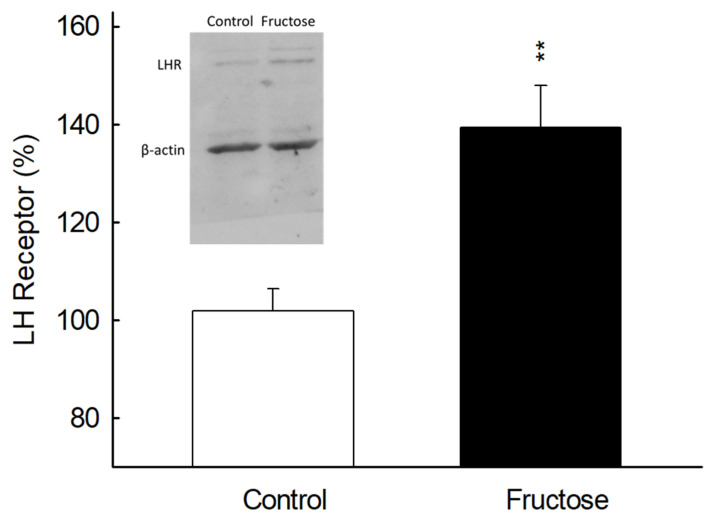
The expression of LH receptors in rat Leydig cells. The protein expression of membrane LH receptors in Leydig cells purified from the control group (white column)and fructose-diet fed group (black column) was examined by Western blot analysis. The intensities of LH receptor bands were adjusted to β actin. Each value represents mean ± SEM. ** *p* < 0.01 as compared to the control group. Similar results were repeated twice.

The protein expression of LH receptors on rat Leydig cells was examined and evaluated by Western blot analysis. The results indicate that LH receptor protein expression was increased in the fructose-fed group as compared to the control group (Figure 7). These experimental results further confirm that the increased testicular sensitivity can be attributed to low blood LH concentrations.

## 4. Discussion

The consumption of fructose-rich foods results in the development of metabolic syndrome features and therefore diabetes [23]. Testosterone deficiency and the failure of the male reproductive system are common clinical features (approximately 25~40%) in T2DM, which is also called diabetic testicular disorder [24]. However, the mechanisms underlying diabetes’ detrimental effects on the male reproductive system are still unclear. Our current study showed that the metabolic syndrome animal model, induced by the high-fructose diet, is associated with decreased sperm motility and lower testosterone synthesis. In rat Leydig cells, purified from fructose-fed rats, LH sensitivity was increased (as evidenced through elevation of LH receptors expression and steroidogenic capacity), suggesting that this inhibition is a secondary defect. Concomitant with these findings, the inference is also confirmed by the lower LH level from the anterior pituitary.

Previous studies have demonstrated that metabolic syndrome reduces the total plasma testosterone levels, which in turn impairs sperm production and motility after 12 weeks of a high-fat diet [25]. Moreover, diabetes significantly affects several sperm parameters, including increased testicular DNA damage and sperm degradation due to alternations in glucose transport and increased oxidative stress [26,27]. In diabetic men, hyperglycemia increases oxidative stress, a common pathology seen in approximately 50% of all infertile men, thereby causing infertility [28]. The inhibition of hyperglycemia on sperm motility was also confirmed by our study.

Elevated fructose consumption is considered as one of the riskiest lifestyles as part of the Western-diet-related diabetes model [29]. Energy depletion and chronic inflammatory responses may lead to testicular disorders and reduced spermatogenesis [30]. With eight weeks of fructose consumption, rats’ testes displayed a morphology disorder and an increase in the apoptosis rate [31]. Our study shows an increase in sperm death and a reduction in testosterone levels. Testosterone secretion not only plays an important role in the male reproduction system, but also in the modulation of insulin secretion [32]. The disturbance of insulin secretion was commonly seen in a high-fructose diet, which could cause abnormal sperm morphology and dysfunction [33].

Male reproductive activities such as sexual behavior, spermatogenesis, and fertility are regulated largely by the hormones of the HPG axis. The synthesis of testosterone, the hormone that plays a vital role in regulating sexual behavior and increasing muscle mass, is mainly regulated by the activation of LH receptors, thereby acting on the cAMP/PKA pathway, and thus elevating the expression and activity of steroidogenic enzymes [34]. The increased LH receptors may be related to the reduction in LH released by the regulation of extracellular signal-regulated protein kinases 1 and 2 (ERK 1/2) [35]. In our current study, the testosterone release in response to hCG in the fructose diet-fed group was dramatically elevated compared to the control group. Additionally, testosterone release induced by 8-Br-cAMP and forskolin was also elevated, although this result was nonsignificant. Similar results were also found in the steroidogenic enzyme activity. These results may be explained by the findings of Purvis et al.’s study, which stated that “when the receptors for LH/hCG are reduced by more than 30% of their normal levels, the sensitivity of the response to LH/hCG is significantly reduced” [36]. In our current study, high-fructose-diet-induced diabetic rats showed a significant reduction in both plasma LH and testosterone levels. Meanwhile, basal LH secretion ability from the anterior pituitary isolated from the fructose-fed group was increased. In contrast, release from the anterior pituitary in response to GnRH was reduced. Moreover, the response of the Leydig cells to upstream hormones caused a significant increase. These results imply that diabetes can abolish the HPG axis, at least in part, through eliminating the negative feedback loop and thereby reducing the production of androgen.

The present study had some limitations. First, we did not measure plasma GnRH levels in the hypothalamic–hypophyseal portal system, which connects the hypothalamus to the anterior pituitary and regulates hormone secretion from the pituitary. Secondly, we did not determine the protein expression of steroidogenic acute regulatory (StAR) protein and cholesterol side-chain cleavage (P450scc), which are the two proteins of the rate-limiting step in steroidogenesis. Therefore, despite the fact that high-fructose feeding affects LH secretion and promotes increased sensitivity of Leydig cells, leading to increased testosterone synthesis, we cannot confirm the notion that hypothalamic GnRH secretion is indeed altered. Additional studies are required to determine the GnRH levels in testis tissues of rats fed with a high-fructose diet.

**Figure 8:**
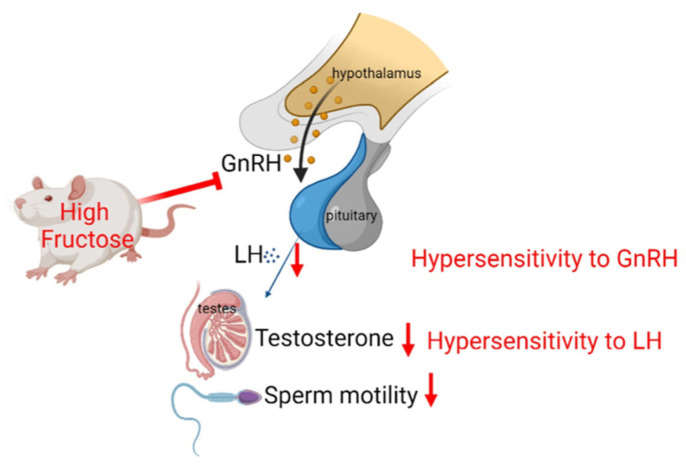
Schematic representation of the mechanisms by which a high-fructose diet impairs male reproductive function following an alternation of the HPG axis.

In summary, the present results demonstrate that the consumption of a high-fructose diet induces metabolic syndrome, thereby abolishing the physiological regulatory function of the HPG axis. Steroidogenesis and sperm motility were reduced in fructose-fed rats. These effects might be due to the inability of the hypothalamus and anterior pituitary gland to appropriately respond to a decline in plasma testosterone levels (Figure 8).

## 5. Conclusions

In this study, we demonstrated that a high-fructose diet decreased plasma testosterone levels and altered the steroidogenesis, which may cause spermatogenesis dysfunction and increase the sensitivity of LH, increasing the risk of male infertility.

## Data Availability

The data presented in this study are available upon request from the corresponding author. The data are not publicly available due to patent application.
